# Cxcl8b and Cxcr2 Regulate Neutrophil Migration through Bloodstream in Zebrafish

**DOI:** 10.1155/2017/6530531

**Published:** 2017-05-31

**Authors:** Constanza Zuñiga-Traslaviña, Karina Bravo, Ariel E. Reyes, Carmen G. Feijóo

**Affiliations:** ^1^Departamento de Ciencias Biologicas, Facultad de Ciencias Biologicas, Universidad Andrés Bello, Republica 217, 8370146 Santiago, Chile; ^2^Interdisciplinary Center for Aquaculture Research (INCAR), Concepción, Chile

## Abstract

Neutrophils play an essential role during an inflammatory response, which is dependent on their rapid recruitment from the bone marrow to the vasculature. However, there is no information about the molecular signals that regulate neutrophil entry to circulation during an inflammatory process in humans. This is mainly due to the lack of a suitable model of study that contains similar set of molecules and that allows in vivo analyses. In this study, we used the zebrafish to assess the role of Cxcl8a, Cxcl8b, and Cxcr2 in neutrophil migration to blood circulation after injury. Using Tg(BACmpx:GFP)^i114^ transgenic embryos and two damage models (severe and mild), we developed in vivo lack of function assays. We found that the transcription levels of *cxcl8a*, *cxcl8b*, and *cxcr2* were upregulated in the severe damage model. In contrast, only *cxcr2* and *cxcl8a* mRNA levels were increased during mild damage. After knocking down Cxcl8a, neutrophil quantity decreased at the injury site, while Cxcl8b decreased neutrophils in circulation. When inhibiting Cxcr2, we observed a decrease in neutrophil entry to the bloodstream. In conclusion, we identified different functions for both Cxcl8 paralogues, being the Cxcl8b/Cxcr2 axis that regulates neutrophil entry to the bloodstream, while Cxcl8a/Cxcr2 regulates the migration to the affected area.

## 1. Introduction

Neutrophils are the most abundant types of leukocytes and neutrophil migration represents the hallmark of inflammation. Under homeostatic conditions, in humans as well as in other mammals, the great majority of neutrophils are retained in the bone marrow and only a small fraction is present in peripheral blood [1]. Under a stress condition, when an inflammatory process is triggered, this fraction rapidly increase ensuring proper response [2]. On the other hand, in several human inflammatory diseases, such as chronic obstructive pulmonary disease, cystic fibrosis syndrome, rheumatoid arthritis, and atherosclerosis, the excessive accumulation of neutrophils in the blood vessels can have deleterious effects. Therefore, it is crucial to precisely control neutrophil levels in the blood to ensure efficiency during wound or infection but at the same time prevent an enhanced response that could damage tissue which would worsen the situation. Although neutrophil migration by circulation is a critical step during an inflammatory process, there is no detailed information about the molecular signals that regulate this process in humans.

In mice, during homeostatic conditions, bone marrow neutrophil retention signals are favored because the CXCL12/CXCR4 pathway is dominant to the promigratory pathway mediated by CXCL1-CXCL2/CXCR2 [3–7]. On the other hand, when an aggression is produced, the levels of promigratory cytokines CXCL1 and CXCL2 increase, displacing the balance towards the migration, thereby increasing the amount of neutrophils that travel from the hematopoietic tissue to the bloodstream. In humans, the primary ligand of CXCR2 is CXCL8, which gene is not present in the mouse genome. Also, humans have a second CXCL8 receptor, CXCR1, absent in mice neutrophils [7, 8]. Therefore, the difference between humans and rodents regarding CXCL8 represents a considerable obstacle, especially when considering that CXCL8 greatly contributes to several chronic diseases in which neutrophils are involved [9–12]. Consequently, it is of utmost importance to identify a suitable biological model that contains the CXCL8/CXCR2 axis and that allows in vivo analyses at the cellular and molecular levels to better understand the molecular signals that regulate inflammation in humans.

In the last decade, zebrafish (*Danio rerio*) have been increasingly used to study innate immunity, particularly in regard to neutrophil functions. As in humans, this teleost fish contains Cxcr1, Cxcr2, and Cxcl8 (found as paralogues Cxcl8a and Cxcl8b) [13, 14]. Also, under normal conditions, the majority of neutrophils are present at the hematopoietic tissue; they are immobile and retained there by the action of the Cxcl12a-Cxcr4 signaling pathway [15]. Therefore, zebrafish may represent a suitable model for understanding which chemokines regulate neutrophil migration by the bloodstream, a process likely to overlap with that present in humans. Previously, we determined that the inflammatory process triggered by severe damage, such a caudal fin transection, differs in several aspects from mild damage, such as a fin cut [16]. For example, in a severe damage model, first-responding neutrophils migrate across the interstitial tissue to reach the wound. Later, neutrophils start to migrate to the damaged tissue by circulation [16]. On the contrary, in a mild damage model, neutrophils only migrate to the affected area through the interstitial tissue. Likewise, the Gcsf-Chr19 cytokine is only upregulated in the severe damage model, acting as an important promoter of neutrophil entry to blood vessels [16], which is similar to the role played by its mammalian orthologue, GCSF [1].

Considering the unique tools available in zebrafish that permit coupling live images of specific, fluorescently labeled cell types with molecular strategies to manipulate gene functions [17–20], the aim of this study was to understand the roles of Cxcr2, Cxcl8a, and Cxcl8b during neutrophil migration by the bloodstream. To achieve this, a series of molecular and pharmacological approaches were used to analyze their participation in vivo. Severe and mild damage models were compared to differentiate the signals that control neutrophil entrance into blood circulation from those governing other steps of the inflammation process, such as final migration to the damaged area. The results obtained indicate that Cxcl8b and Cxcr2 are key regulators of neutrophil migration by the bloodstream in zebrafish.

## 2. Materials and Methods

### 2.1. Zebrafish Strains and Maintenance

Zebrafish were maintained and raised according to standard protocols [20]. The following strains of fish were used in this study: Tg(BACmpx:GFP)^i114^ [21] and Tg(fli1a:EGFP)^y1^ [22]. All embryos were collected through natural spawning, staged according to Kimmel et al. [23], and raised at 28°C in Petri dishes containing the E3 medium (5 mM NaCl, 0.17 mM KCl, 0.33 mM CaCl_2_, 0.33 mM MgSO_4_, with methylene blue 0.01%, and equilibrated to pH 7.0), as previously described in Westerfield et al. [20]. Embryonic and larval ages were expressed as hpf or dpf. All damage experiments were performed at 48 hpf. All maintenance and experimental protocols were reviewed and approved by the Animal Ethics Committee of the Universidad Andrés Bello to ensure animal welfare.

### 2.2. Damage Models

**Figure 1:**
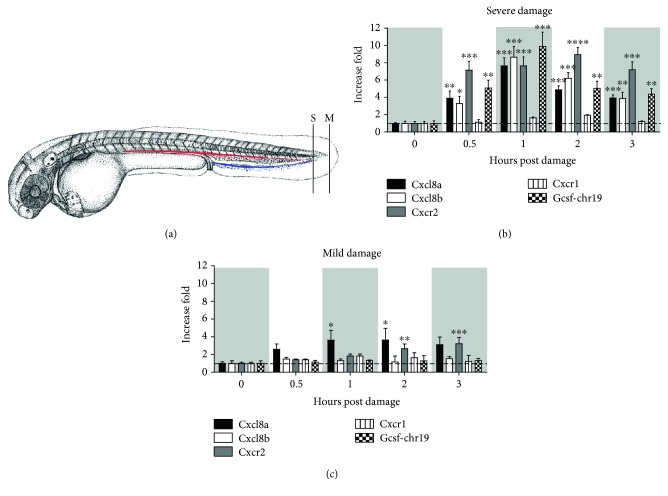
Severe and mild damage differentially regulate the transcription of cxcl8 paralogues in zebrafish. (a) Diagram showing the location of severe (S) and mild (M) damage on the caudal region and caudal fin of the embryo, respectively. The red line corresponds to the caudal artery, and the blue line to the caudal vein. (b, c) Transcription levels of *cxcl8a*, *cxcl8b*, *cxcr2*, *cxcr1*, and *gcsf-chr19* were quantified by qPCR after (b) severe or (c) mild damage. Data are presented as fold of change over each level at 0 hours post damage and normalized to *b-actin1*. ^∗^*p* value < 0.05; ^∗∗^*p* value < 0.01; ^∗∗∗^*p* value < 0.005.

Previous to receiving any injury, embryos were anesthetized with 0.017% tricaine [24]. For the mild damage model, the caudal fin, excluding muscle, was transected. For the severe damage model, the protocol described by Elks et al. [24] for caudal fin transection was followed. This damage model included a small section of muscle from the most caudal end of the embryonic body (Figure 1(a)). All injuries were performed on 56–58 hpf Tg(BACmpx:GFP)^i114^ or Tg(BACmpx:GFP)^i114^ X Tg(fli1a:EGFP)^y1^ transgenic embryos. In the latter case, at this stage, no more green myeloid cells were seen in the Tg(fli1a:EGFP)^y1^. For Tg(BACmpx:GFP)^i114^ X Tg(fli1a:EGFP)^y1^ transgenic fish, embryos with 2-3 disruptions in the intersegmental vessels, but with no defect in the dorsal artery or caudal vein, were selected to ensure that neutrophils could travel through the main vessels of the embryo to reach the target destination (Supplementary Figure 1 available online at https://doi.org/10.1155/2017/6530531).

### 2.3. Neutrophil Quantification

Neutrophils in the dorsal and damaged area were quantified according to the computational method described by Ellet and Lieschke [25]. Following this method, Tg(BACmpx:GFP)^i114^ or Tg(BACmpx:GFP)^i114^ X Tg(fli1a:EGFP)^y1^ transgenic larvae were photographed, and every picture was analyzed using the ImageJ software. Quantification was measured in leukocytes units (LEU) or the percentage of neutrophils present in the damaged tissue in relation to the total amount of neutrophils in the larval tail. Neutrophils in blood circulation were quantified in the posterior cardinal vein of each embryo using a 5 min movie with 4 s intervals in the Cxcr2 inhibition experiments. For double transgenic experiments microinjected with Cxcl8a and Cxcl8b morpholino (MO), the neutrophils in circulation were quantified in the caudal vein using a 5 min movie with 10.5 s intervals in each embryo.

### 2.4. Knockdown Experiments

Both morpholino MO5-cxcl8a (from now on Cxcl8a MO) and MO1-cxcl8b.1 (from now on Cxcl8b MO) used in the present study were previously used and proved to be effective and efficient in inhibiting the splicing of their corresponding gene [26]. The corresponding sequences are shown in Table 1. Each embryo was injected with 8 ng of Cxcl8a MO or 20 ng of Cxcl8b MO at the 1-cell stage. The knockdown of *cxcl8a* and *cxcl8b* was confirmed through RT-PCR (Supplementary Figure 2).

### 2.5. RT-qPCR

Total RNA was extracted from 40 embryos at 0, 0.5, 1, 2, and 3 hours post performing mild or severe damage. Total RNA was extracted using the TRIzol Reagent (Invitrogen) according to the manufacturer's instructions. The cDNAs were synthesized from RNA samples with a reverse transcription reaction that used oligo-dt primers and SuperScript II RT (Invitrogen) according to the manufacturer's instructions. Real-time PCR conditions were as follows: 40 cycles at 94°C for 30 s, 59°C for 25 s, and 72°C for 30 s. Each gene was tested, and the melting curves were verified. The mean Ct values from each sample were normalized against the mean Ct value of a reference gene (*β-actin1*, housekeeping gene). The relative quantification of each gene was obtained with the Pfaffl method [27]. The primers used are shown in Table 2.

### 2.6. Cxcr2 Inhibition Experiments

Experiments with SB225002 (Cxcr2 inhibitor) were performed as previously described by Deng et al. [28]. Zebrafish embryos were preincubated 30 min before caudal fin transection with 5 *μ*M of SB225002 (Calbiochem, EMD Millipore) in the E3 medium with 1% dimethyl sulfoxide. The embryos were maintained in this solution after fin transection over the entire course of the experiment.

### 2.7. Statistics and Imaging

**Figure 2:**
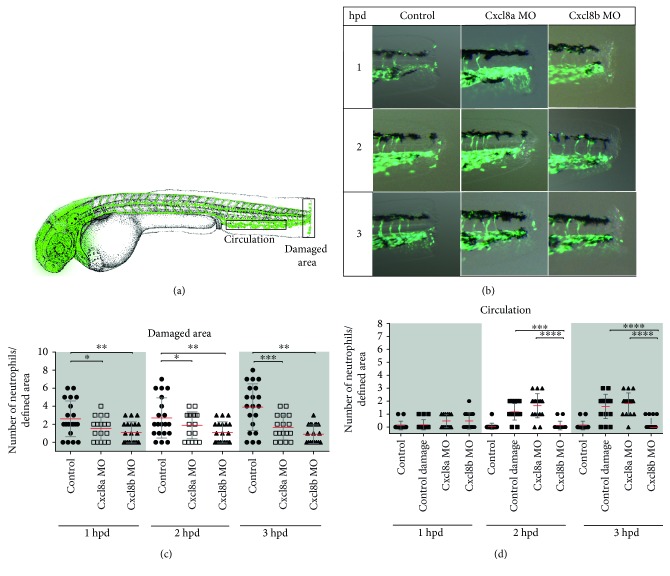
Neutrophil migration decreases when Cxcl8a or Cxcl8b are inhibited during severe damage. (a) Diagram showing the quantified neutrophils in two areas. (b) Lateral view of the caudal section of the embryo tail at 1, 2, and 3 hpd in control and morphant embryos. (c) Quantified neutrophils at the damaged area at 1, 2, and 3 hpd. (d) Quantified neutrophils in circulation during homeostasis, after severe damage and in the absence of Cxcl8a or Cxcl8b function. The neutrophils were quantified at 1, 2, and 3 hpd. ^∗^*p* value < 0.05; ^∗∗^*p* value < 0.01; ^∗∗∗^*p* value < 0.001; ^∗∗∗∗^*p* value < 0.0001.

**Figure 3:**
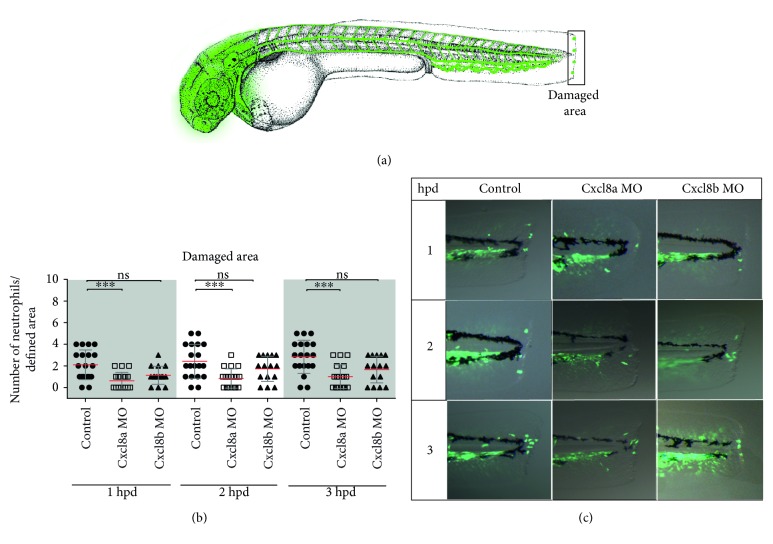
Neutrophil migration decreases when Cxcl8a, but not Cxcl8b, is inhibited during mild damage. (a) Diagram showing the quantified neutrophils at the wound area. (b) Quantified neutrophils at damaged area at 1, 2, and 3 hpd. (c) Lateral view of the caudal section of the embryo tail at 1, 2, and 3 hpd in the control and morphant embryos. ^∗∗∗^*p* value < 0.005.

**Figure 4:**
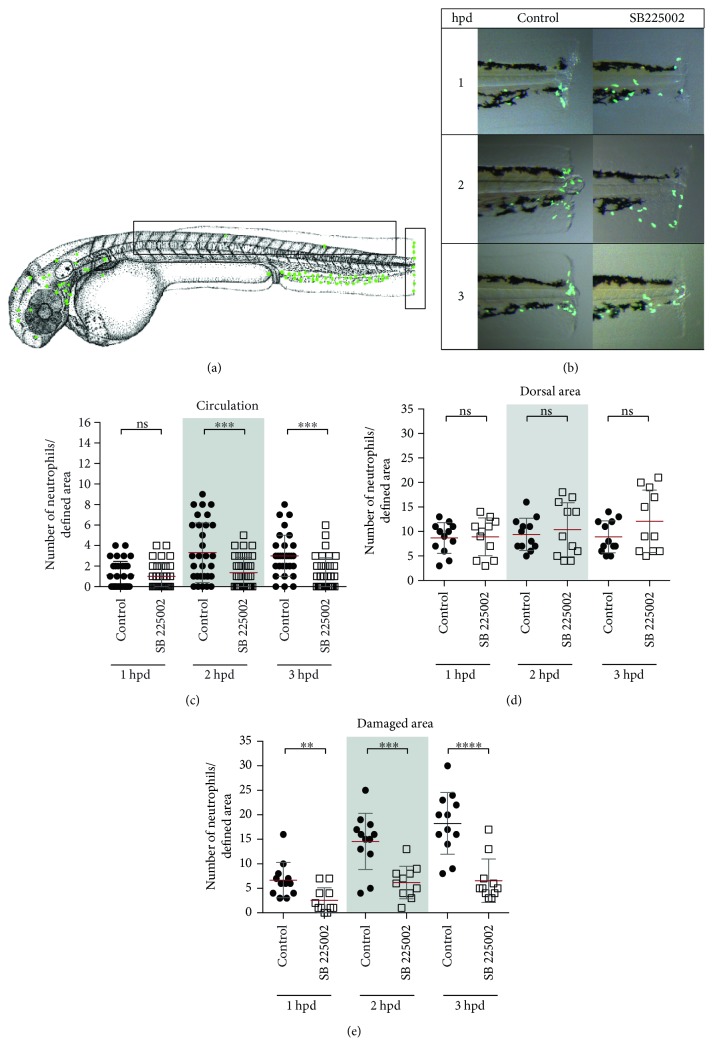
Cxcr2 inhibition decreases neutrophil entrance to the bloodstream and tissue infiltration in severe damage. (a) Diagram showing the quantified neutrophils, in circulation and at the dorsal and damaged area. (b) Neutrophils in circulation at 1, 2, and 3 hpd in control embryos and treated with the inhibitor SB225002. (c) Lateral view of the caudal section of the embryo tail at 1, 2, and 3 hpd in the control and treated embryos. (d, e) Quantified neutrophils at the dorsal and damaged areas at 1, 2, and 3 hpd. ^∗∗^*p* value < 0.01; ^∗∗∗^*p* value < 0.005; ^∗∗∗∗^*p* value < 0.0001.

**Figure 5:**
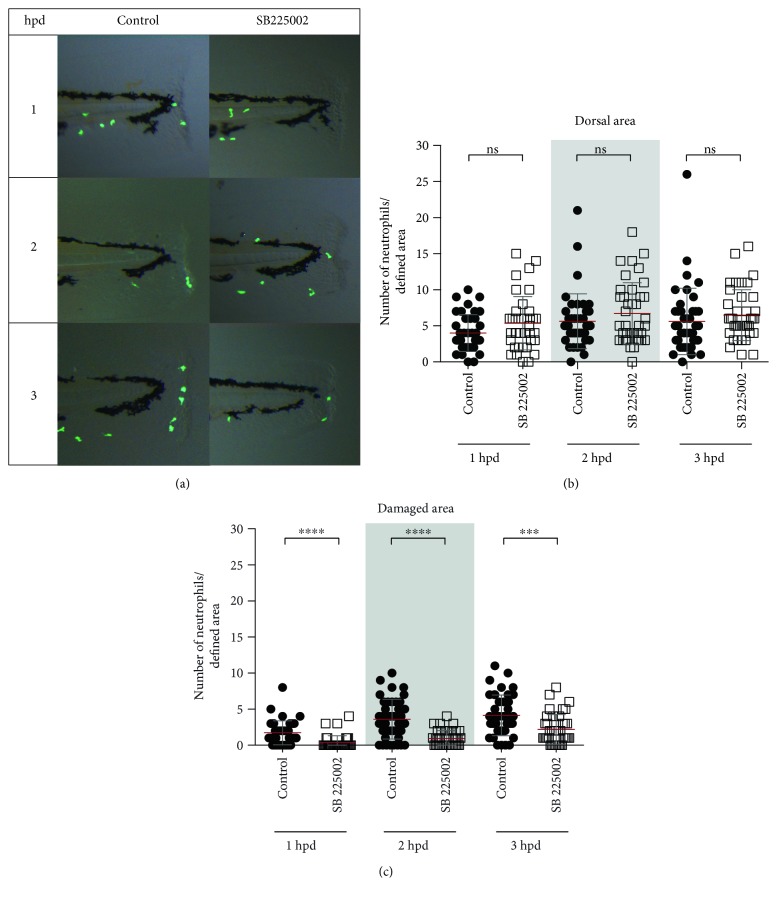
Cxcr2 inhibition decreases neutrophil infiltration in mild damage. (a) Lateral view of the caudal section of the embryo tail at 1, 2, and 3 hpd in the control and treated embryos. Demarked with a white rectangle are the two quantified areas. (b, c) Quantified neutrophils at the dorsal and damaged areas at 1, 2, and 3 hpd. ^∗∗∗^*p* value < 0.005; ^∗∗∗∗^*p* value < 0.0001.

In the case of qPCR (Figure 1), 40 individual were used for RNA extraction and RT-qPCR; the data showed is from one representative experiment from at least three biological replicates. Likewise, for the in vivo experiments (Figures 2, 3, 4, and 5), at least 20 individuals were included in each assay and three biological replicates were performed. In Figures 1, 2, and 3, data were analyzed using nonparametric Kruskal-Wallis, two-way ANOVA, and Dunn's multiple comparison tests. The data were normally distributed (analyzed by the D'Agostino-Pearson normality test), but variance was not homogenous (analyzed by the Brown-Forsythe test). For Figures 4 and 5, data were analyzed with the nonparametric test Mann–Whitney. All analyses were performed using Prism 6 (GraphPad Software), and the significance level was set at *P* < 0.05. Photographs were taken in an Olympus SZX16 stereoscope with the QImaging MicroPublisher 5.0 RVT camera. Images were processed with Photoshop CS5 or ImageJ 1.44o [29]. All of the described experiments were performed at least three times, and the images shown are representative of the effects observed in at least 70% of the individuals.

## 3. Results

### 3.1. Severe Damage Upregulates *cxcl8a*, *cxcl8b*, *gcsf-chr19*, and *cxcr2*, While Mild Damage Only Upregulates *cxcl8a* and *cxcr2*

To determine the roles of Cxcl8a (previously named Cxcl8l1 [14] and zCxcl8 [30]), Cxcl8b (previously named Cxcl8l2 [14]), and Cxcr2 in neutrophil migration through the bloodstream during mechanical damage, the transcriptional levels of these genes were determined in vivo using severe and mild damage models, taking into consideration the differences in the inflammatory processes generated by each type of injury [16]. As a control of the type of damage generated, the mRNA levels of *gcsf-chr19*, a critical cytokine for neutrophil blood vessel entry, were assessed [16]. Severe damage increased the mRNA levels of *cxcl8a*, *cxcl8b*, *gcsf-chr19*, and *cxcr2*. Specifically, as early as 30 minutes after severe damage, all of these molecules were upregulated, with peak expression occurring 1 hpd before slowly declining, reaching normal levels at 3 hpd (Figure 1(b)). On the other hand, mild damage only increased the transcription of *cxcl8a* and *cxcr2* (Figures 1(b) and 1(c)). Furthermore, *cxcl8a* and *cxcr2* upregulation was delayed in comparison with the severe damage model, starting at 1 hpd for *cxl8a* and at 2 hpd for *cxcr2* (Figure 1(c)). There was no increase in the mRNA levels of *cxcr1* during the entire time course for either severe or mild damage.

### 3.2. Cxcl8a Knockdown Decreases Neutrophil Quantity at the Injury, While Cxcl8b Decreases Neutrophils in Circulation

Considering the transcriptional differences observed in the qPCR analysis for *cxcl8a* and *cxcl8b* between the severe and mild damage models, the functions of both genes were inhibited by MO and the effects of this on neutrophil migration to the wound were determined in vivo. Since Cxcl8 plays an important role in angiogenesis, particularly in intersegmental vessel formation [31], Cxcl8a MO or Cxcl8b MO was microinjected in double transgenic embryos, Tg(BACmpx:GFP)^i114^ X Tg(fli1a:EGFP)^y1^, to correctly identify morphant embryos (Supplementary Figure 1). Both neutrophils and blood vessels of double transgenic fish are fluorescently labeled.

In the severe damage model, the absence of either Cxcl8a or Cxcl8b significantly decreased the amount of neutrophils present at the wound in comparison with that of control-damage embryos, in which the amount of neutrophils present at the damaged area continuously increased over the time course trial (Figures 2(b) and 2(c)). In addition, neutrophils in blood circulation were quantified (Figure 2(d)). In control-damage embryos, neutrophils were still high in circulation at 3 hpd (Video 1), in contrast to the noninjured control embryos that lack neutrophils in the bloodstream (Videos 2 and 3). No differences were observed between MO-injected Cxcl8a and control-damage fish. Remarkably, severely damaged morphant embryos for Cxcl8b showed no neutrophils in circulation, just as observed in the noninjured control embryos. On the other hand, in the mild damage model, only the absence of Cxcl8a affected the quantity of neutrophils at the wound. The amount of neutrophils that reached the wound in Cxcl8b morphant embryos presented no significant difference with control-damage embryos (Figure 3). Thus, the results obtained through in vivo analysis using MOs to block the functioning of each Cxcl8 paralogue were consistent with qPCR analyses and suggest different functions for Cxcl8a and Cxcl8b.

### 3.3. Pharmacological Inhibition of Cxcr2 Decreases the Amount of Neutrophil in the Bloodstream

To analyze Cxcr2 participation in neutrophil migration trough circulation, its function was pharmacologically inhibited, using the specific inhibitor SB225002. White and collaborators [32] demonstrated that SB225002 is a potent and selective nonpeptide inhibitor of Cxcr2, both in vitro and in vivo. Thus, in the severe damage model, we quantified neutrophil number in circulation and in damaged area and included a third area (dorsal area) as a nonspecific region (Figure 4(a)). The results obtained showed that the number of neutrophils present at the dorsal area was not different from that observed in control-damage embryos (Figure 4(d)). In contrast, the amount of neutrophils detected in the bloodstream of inhibitor-treated embryos was significantly lower than that of control-damage embryos at least until 3 hpd (Figure 4(c), Videos 4 and 5). Likewise, the number of neutrophils that reached the injury site was lower in inhibitor-treated embryos than that in controls during the entire time course trial (Figures 4(b) and 4(e)). In the mild damage model (Figure 5), the number of neutrophils present at the damaged area in inhibitor-treated embryos was drastically lower at each of the analyzed time points (Figures 5(a) and 5(c)). In contrast, the number of neutrophils detected in the dorsal area of inhibitor-treated embryos showed no difference compared with that of controls (Figure 5(b)).

## 4. Discussion

Neutrophils are the first cells to be recruited to a site of infection or damage, and neutrophil migration is regulated by different chemokines. However, which chemokines and receptors involved in the regulation of these leukocytes' migration into blood circulation is unknown in both humans and, prior to this study, zebrafish. By using a series of methodological approaches and two different models of damages (severe and mild), a role for Cxcl8b and Cxcr2 in neutrophil entry to bloodstream was identified. Similarly, it was determined that Cxcl8a, but not Cxcl8b, attracts neutrophils to the wound area. Taken together, these data provide the first functional characterization of neutrophil migration by bloodstream after mechanical damage in zebrafish.

The transcriptional analysis showed that in a severe damage model, all the genes analyzed are increased, suggesting the participation of all of them in the inflammation process. On the other hand, during the mild damage, only *cxcr2* and *cxcl8a* were upregulated, implying that some events occurring during the severe damage are not activated in this situation. One of these events is neutrophil migration by blood circulation, thus suggesting that Cxcl8a is only involved in the chemoattraction of neutrophils through the extracellular matrix. The lack of function assays developed confirmed these results. In the absence of Cxcl8a, the number on neutrophils present in the bloodstream is indistinguishable from control-damage embryos. In a preliminary analysis, it seems that these results do not agree with those obtained by the group of De Oliveira [26]. In their work, they indicate that the absence of either Cxcl8a or Cxcl8b decreases the number of neutrophils that reach the wound and conclude that both chemokines regulate neutrophil migration to the injury site. We observe the same in our severe damage model, the lack of each Cxcl8 paralogue affects the number of neutrophils that arrive at the wound area, but only the absence of Cxcl8b decreases the number of circulation neutrophils. Thus, we agree that the lack of each Cxcl8 paralogue affects the final number of neutrophils that reach the damage, but we think that the process altered in each case is different suggesting that Cxcl8a and Cxcl8b regulate different steps of the neutrophils' journey to the inflamed site. Also, they should be expressed in different tissues, Cxcl8a at the wound and Cxcl8b at the endothelium near the CHT.

On the other hand and although the function of CXCL8 in neutrophil entry to circulation is not clear in humans, the function of CXCL8 in a similar process, such as neutrophil extravasation, is well documented [33–36]. During neutrophil transendothelial migration, glycosaminoglycan-immobilized CXCL8 at the luminal surface of endothelial cells allows neutrophil adhesion and posterior emigration to surrounding tissue. This mechanism could shed light onto how neutrophils enter the bloodstream. Considering this and the present results regarding Cxcl8b, the CXCL8-endothelial cells-neutrophils interaction could also function in the opposite direction. In other words, zebrafish Cxlc8b could be immobilized and exposed to the abluminal endothelial surface, thereby allowing neutrophil contact with and entrance to the vasculature. Indeed, the entry of neutrophils to blood circulation occurs not only at the begging of the inflammatory process but also during resolution by reverse migration, a process that has been observed in vitro and in vivo in zebrafish and mice [37–41].

The function exerted by CXCL8 on neutrophils in humans can be divided into roles related to the vasculature and to the interstitial tissue. In zebrafish, CXCL8 orthologues contribute to both functions, but each role is performed by a separate paralogous gene, Cxcl8a or Cxcl8b. The existence of two orthologous CXCL8 genes in zebrafish is attributable to the genome duplication event that occurred near the base of the ray-finned fish evolutionary tree [42]. Indeed, the repertoire of chemokines present in zebrafish is twice that of humans (89 and 44, resp.) [43, 44].

On the other hand, in the current study, Cxcr2 was found to participate not only in the final neutrophil migration to the wound but also in neutrophil migration through the bloodstream. It is interesting that in the Cxcr2 lack of function assay, a low amount of neutrophils still circulate, suggesting that another chemokine receptor could also participate in the process but to a lesser extent. This is supported by the fact that in the Cxcl8b lack of function assay, no neutrophil was detected in the bloodstream. A receptor that is a good candidate to be involved in this process is Cxcr1, mainly because it interacts with CXCL8 in humans [8]. Finally, and not expected, we found that there is a neutrophil subpopulation that after injury migrates through the interstitial tissue in a Cxcr2-independent form. Moreover, these neutrophils did not migrate in wound direction but to the dorsal area and could or not be found later at the injury site.

The participation of CXCR2 in bone marrow neutrophil release is documented in mice, where neutrophils lacking CXCR2 are preferentially retained in the bone marrow, causing chronic neutropenia [7, 9, 45–47]. Several studies support the hypothesis that neutrophil release is antagonistically regulated by the CXCR2 and CXCR4 chemokine receptor system [7, 48]. Under homeostatic conditions, neutrophil retention signals are favored in the bone marrow since the CXCL12/CXCR4 pathway is dominant to the promigratory pathway mediated by the CXCR2/CXCL1-2 axis. When neutrophil release from the hematopoietic tissue is required, the levels of the promigration cytokines CXCL1 and CXCL2, as well as G-CSF, increase, thereby displacing the balance towards migration [7]. In a previous study, we demonstrated that zebrafish Gcsf-Chr19 regulates neutrophil migration by the bloodstream after mechanical damage [16]. In turn, the present study provided new details for how neutrophils are mobilized from the caudal hematopoietic tissue to the circulation after a sterile stimulus by addressing the role of Cxcr2 in this process and by confirming the evolutionary conservation of Cxcr2 function in lower vertebrates, such as fish. Furthermore, the present results suggest that Cxcr2 is the receptor for both Cxcl8 paralogues Cxcl8a and Cxcl8b.

**Figure 6:**
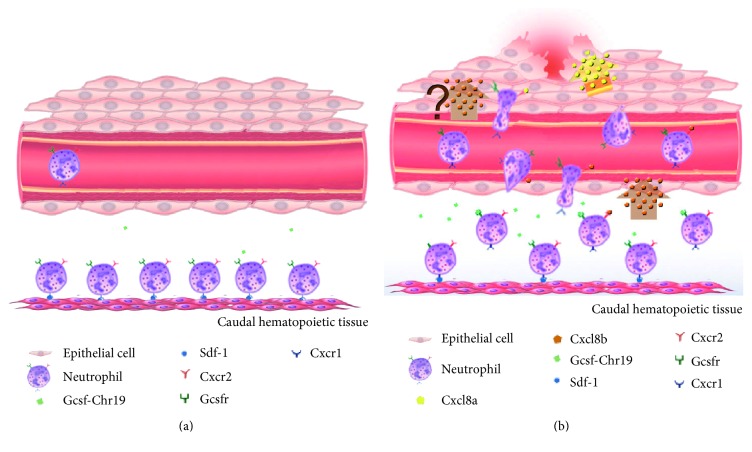
Model for the regulation of neutrophil migration to a wound. (a) During homeostasis, neutrophils are retained in the caudal hematopoietic tissue by Cxcr4/Cxcl12. (b) After damage, the expression of Gcsf-Chr19 would increase and interact with its receptor, Gcsfr. Likewise, mRNA levels of Cxcl8b and Cxcr2 would increase, and both proteins would interact. Therefore, both signaling pathways would allow neutrophils to leave the caudal hematopoietic tissue and enter the bloodstream. Neutrophils would stay in circulation until sensing an unknown signal (probably Cxcl8b) in the endothelium, then leaving the blood vessel. Finally, in the interstitial tissue, Cxcl8a/Cxcr2 would guide neutrophils to the wound.

In conclusion, and by consolidating previous and our present data, Cxcl8b and Cxcr2 are key regulators of neutrophil entrance into blood circulation in zebrafish. In more detail, we propose the following model regarding neutrophil migration during an inflammatory process in zebrafish (Figure 6). During homeostasis, neutrophils are retained in the caudal hematopoietic tissue by Cxcr4/Cxcl12 [49], meaning only a few neutrophils would be in the bloodstream (Figure 6(a)). After severe damage (Figure 6(b)), Gcsf-Chr19, Cxcl8b, and Cxcr2 expression would increase, and Cxcl8b would bind to Cxcr2. Considering the overexpression of Gcsf-Chr19, it is plausible to hypothesize that this molecule would functionally interact with its receptor, Gcsfr. Therefore, both the Cxcl8b/Cxcr2 and Gcsf-Chr19/Gcsfr signaling pathways would allow neutrophils to leave the caudal hematopoietic tissue and enter the bloodstream [16]. This would induce neutrophils to enter and remain in circulation until sensing an unknown signal (probably Cxcl8b) in the endothelium near the site of injury, where neutrophils would then leave the blood vessels. Furthermore, in the interstitial tissue, Cxcl8a would bind to Cxcr2 present in neutrophils to enable neutrophils to reach the wound [26, 28, 50].

Our results significantly contribute to fill the gap regarding the molecular signals that regulate inflammation and neutrophil recruitment from the hematopoietic tissue to the vasculature in zebrafish, a key step of the journey of this granulocyte during an inflammatory process. Considering the similarity in molecules between zebrafish and humans—which made this fish a suitable model for this study—our research provides new avenues for understanding neutrophil biology during homeostasis and pathologic conditions.

## Supplementary Material

The information of supplementary materials are as follows: Supplementary Figure 1: Morphant phenotype. (A, B, C) Representative images of the different phenotypes observed after Cxcl8a or Cxl8b morpholino microinjection. (A) Control phenotype showing vasculature disruption. (B) Morphant phenotype used in the damage assays, showing 2-3 disruptions only in intersegmental vessels (arrow). (C) Severe phenotype, with the vasculature showing more than three alterations. Supplementary Figure 2: RT-PCR showing the effect of the MO5-cxcl8a and MO1-Cxcl8b.1 on its respective transcript splicing. RNA prepared from embryos injected with control MO shows the internal control band (b-actin) and the correct spliced mRNA for Cxcl8a or Cxcl8b. On the contrary, in the case of both types of morphant embryos a new band with an increased length appeared (arrow), indicating defective mRNA splicing. Video 1: Amount of circulating neutrophils after a severe damage in Tg(BACmpx:GFP)i114 x Tg(fli1a:EGFP)y1 embryos. Zoom of the caudal hematopoietic tissue, showing the passage of 3-4 neutrophils through vasculature. Video 1 tracking: Amount of circulating neutrophils after a severe damage in Tg(BACmpx:GFP)i114 x Tg(fli1a:EGFP)y1 embryos. Zoom of the caudal hematopoietic tissue, highlighting the passage of neutrophils through vasculature (arrow head). Video 2: Amount of circulating neutrophils after a severe damage in Cxcl8b morphant embryos in Tg(BACmpx:GFP)i114 x Tg(fli1a:EGFP)y1 embryos. Zoom of the caudal hematopoietic tissue, showing the passage of only 1 neutrophil through vasculature. Video 2 tracking: Amount of circulating neutrophils after a severe damage in Cxcl8b morphant embryos in Tg(BACmpx:GFP)i114 x Tg(fli1a:EGFP)y1 embryos. Zoom of the caudal hematopoietic tissue, highlighting the passage of 1 neutrophil through vasculature (arrow head). Video 3: Amount of circulating neutrophils after a severe damage in Cxcl8a morphant embryos in Tg(BACmpx:GFP)i114 x Tg(fli1a:EGFP)y1 embryos. Zoom of the caudal hematopoietic tissue, showing the passage of 2-3 neutrophils through vasculature. Video 3 tracking: Amount of circulating neutrophils after a severe damage in Cxcl8a morphant embryos in Tg(BACmpx:GFP)i114 x Tg(fli1a:EGFP)y1 embryos. Zoom of the caudal hematopoietic tissue, highlighting the passage of 2 neutrophil through vasculature (arrow head). Video 4: Amount of circulating neutrophils after a severe damage in Tg(BACmpx:GFP)i114 embryos. Zoom of the caudal vein, showing the passage of several neutrophils through vasculature. Video 4 tracking: Amount of circulating neutrophils after a severe damage in Tg(BACmpx:GFP)i114 embryos. Zoom of the caudal hematopoietic tissue, highlighting the passage of 6 neutrophils through vasculature (arrow head). Video 5: Amount of circulating neutrophils after a severe damage in Tg(BACmpx:GFP)i114 embryos treated with Cxcr2 inhibitor. Zoom of the caudal vein, showing the passage of few number neutrophils through vasculature (arrow head). Video 5 tracking: Amount of circulating neutrophils after a severe damage in Tg(BACmpx:GFP)i114 embryos treated with Cxcr2 inhibitor. Zoom of the caudal hematopoietic tissue, highlighting the passage of 1 neutrophil through vasculature (arrow head).

## Figures and Tables

**Table 1:** Morpholino sequences.

Gene	Sequence 5′ → 3′	Concentration
Cxcl8a	GGTTTTGCATGTTCACTTACCTTCA	10 ng/embryo
Cxcl8b	TTAGTATCTGCTTACCCTCATTGGC	20 ng/embryo

**Table 2:** Primers sequences.

Gene	Primer	Sequence 5′ → 3′
*β*-Actin1	Forward	TTCTGGTCGTACTACTGGTATTGTG
	Reverse	ATCTTCATCAGGTAGTCTGTCAGGT
Gcsf-chr19	Forward	GTGAGTTCCAGATCCCGACG
	Reverse	TGTGATGAAGCTCCAGACCG
Cxcl8a	Forward	TGTGTTATTGTTTTCCTGGCATTT
	Reverse	GCGACAGCGTGGATCTACAG
Cxcl8b	Forward	CTACCGAGACGTGGGTGATT
	Reverse	GCTCGGTGAATGGTCATTTT
Cxcr2	Forward	TGACCTGCTTTTTTCCCTCACT
	Reverse	TGACCGGCGTGGAGGTA
Cxcr1	Forward	TTCAGTTCGGCTGCACTATG
	Reverse	GGAGCAACTGCAGAAACCTC
